# Rheumatism-Cases

**Published:** 1881-10

**Authors:** C. F. Nichols

**Affiliations:** Boston


### CLINICAL CASES.—RHEUMATISM.

C. F. Nichols, M. D., Boston.

I. 1875, May 10th. Addison N., æt. 50, large, blonde. Was treated for hip disease (left side) in childhood; left leg the shorter.

Rheumatism hereditary from mother. Attacks increasingly severe and frequent since 1860; lately six to eight months apart; on bed or crutches past two years.

The patient lay reeking in sweat, enwrapped like a Dutch child in flannels white and red, and covered by five heavy blankets; the slightest draft of air—even the warm air of the room set in motion by passing attendants—caused chill and aggravated pains. It may as well be noticed here, that the barbarous drainage of sweating is annexed to the treatment of rheumatism with the worst results. Woolens should be removed from *immediate* contact with the skin in all sweating cases, whether rheumatic or phthisical, and also to prevent over-copious sweating. *The sick do not catch cold from this change.* Hence, before presenting the symptoms of this case, we note that he, in common with every other rheumatic since treated by the writer, was made comfortable in a heavy twilled cotton night-dress, whereupon but two blankets were needed, and the outer air blew freely all around him next day, without causing chill.

*Symptoms.*—Pains and swellings wander from joint to joint, knees, ankles, toes and wrists, chiefly left side; swellings pink, hot; knee-joints ecchymose a few hours after intensest pain; pains heavy, grinding (ankles), drawing, shooting, prickling (knees), soles sore. Whenever dry eczema appears on left wrist the pains decrease, worse at change of seasons (spring and fall), during and after motion, from pressure (weight of bed-clothes) all night, while letting legs hang down, from draughts of air (warm or cool), when lying on the left (affected) side. Relieved by hot compresses; must have legs moved frequently, though painful. Restlessness in legs, tosses body. Thirst increases (for large quantities) every evening. Tongue dry, thin brown coat. No appetite, but takes fruit and beef-juice. Pulse 80 to 100. Irascible, despondent, full of fears, especially when alone. Heart-sounds normal. Urine clear, varying acid reaction, red sediment at times, no albumen. Has had no medicine for a week; all sorts previously.

*Arsen.* 200 (D.), in water, was given every three hours, for forty-eight hours. Considerable improvement first three days.

May 18th.—Soles very sore; worse evenings; little fever; tearful. *Puls.* 200, in water, four doses.

Is cross and ugly every morning. Takes no breakfast. Moderately gaining, out of bed, and on crutches until June 15th, when pains wandered about chest, were worse from motion of chest in breathing, felt in knees when moving them. *Bryon.* 200, same dose,

June 22d, *Hepar* 200 (worse from draughts of air, and after sweating). Notes do not show present indications, for the following under which *though better than for some years*, the patient was still on crutches. July 1st, *Lycop.* 200; August 17th, *Sulph.* 6 M; Sept. 5th, *Calc. carb.* 200.

Sept. 14th. (About the time of usual annual attack). Legs stiff, knees swell. Pains dull but constant. Soles very sore. *Lately and suddenly, has noticed an abnormal increase of hair, coarse, dark (hair of head and beard are sandy), long, in tufts, on legs, arms, lower back, etc.* (Says vaccination never took, says he never had gonorrhœa. Warts from time to time). This characteristic of *Thuya* is noted by Wolf, (see " *Allen's Encyclopædia*," *Thuya*, Skin).

Oct. 1st. Has improved every day since taking *Thuya.* Has occasional sharp pains like cramp in right leg. Bunions on joints very sore (no eruption), sometimes feels as if boiling water passed through the bunions. Hairs grow, also increase on genitals. No medicine.

1876, Jan. and June brought slight attacks, neither of which confined him to bed, and he has remained otherwise in good health, with a cane for company when walking.

II. 1876, Sept. 3d. J. B., æt. 22, teamster, plethoric, vigorous. Within four years two attacks of inflammatory rheumatism, with cardiac symptoms; under hospital treatment eight and twelve weeks respectively.

Severe grinding pain in left hip; stitch, when moving or breathing, above left hip extending into lumbar region. Relief while drawing up the leg, and from hot applications. Feet cold, head and body very hot. Little sweat. No thirst. Drowsy. Pulse 120. Heart-sounds uniform, excited and violent, with heaving chest. Was taken sick four hours ago, after working hard.

*Ferrum phos.* 12 centes. trit., in water, every two hours.

Sept. 4th. Pains are less violent, same locality, same conditions. Calls for constant renewal of hot wet cloths. Moves legs restlessly, but less quiet. *Magnes. phos.* 12.

Sept. 5th. No relief from heat. Worse when touched. Very irritable and cross. Given *Bryon.* 200.

Sept. 6th. Says back grows worse. Pain in sacrum. Pain increases if he sits up. Dizzy on sitting up. Better after sleep. Face flushed or pale by turns. *Bell.* 200.

Sept. 8th. Pain to left of sacrum, *severe only while sitting*. *Zinc. met.* 200, dry. Went to work next week.

" Your remedies act well enough upon women and children, but they can't have power to affect a vigorous man!" *Same superstition* in following case:

III. 1880, Dec. 7th. F. S. R., short, tough, dark, proprietor of a stable, where he stands in damp cold. Rheumatism hereditary. Lame left leg, with contracted tendons. Attacks occur in damp weather, lately every two or three months, is out of doors with present attack. Left knee swollen, sensitive to first touch, relieved after gentle rubbing. Worse before a storm, somewhat worse after rest, and again after long standing. Sensation as of a cord slipping inside knee. Chilly all over. Cold legs. Sweats occasionally. Lies on left side. Wakeful after midnight. Syphilis eight years ago.

*Rhus tox.* CM (Swan) in water, four doses, six hours apart. Symptoms had left within thirty-six hours. Did not return during the winter or spring.

IV. 1880, Dec. 9th. Mrs. T., æt. 44, weighs 190. Attacks of articular rheumatism have become increasingly severe and frequent for ten years past. The present attack follows hard work. Pains began yesterday in left hip and left toes, have passed to right shoulder and right knee; heat in these joints, no swelling, pains increase on moving or touching the parts, hot, drawing, twisting "skewer-like," dart through chest. Unrest. Nausea, vomits ingesta. Thirst. Urine spirts when coughing, cough dry, urine turbid, water and bile. When lying on left side, dyspnœa increases. Pulse 110. Heart-sounds irregular, muffled. Very drowsy, but no sleep. *Bryon.* CM, in water every three hours.

Dec. 10th. Weeps, fears death, face pallid, anguished. Pains no better. Thirst and unrest night and day. *Arsen.* CM, same dose.

Dec. 13th. Pulse 100. Urine scanty, infrequent, offensive. Shock in abdomen, and urine spirting from cough. Sleeps with head dropped forward, eyes half open, and jaw down. Sweat only on head. *Pains worse in and after sleep*, stitches in cardiac region *arouse her from sleep*. *Sulph.* DM, dry.

Dec. 15th. Tingling swollen fingers, hands and feet. Pains are chiefly in the right knee, wrists and finger-joints, hot swellings come and go. *Finger and knee-joints dislocate* at times of greatest pain, causing great agony until reset. Must be turned over frequently in bed, *must have legs and arms moved* for temporary relief. Nausea appears, with pains downward in abdomen. Tongue brownest in centre, no coat on the edges. Top of head cold to touch, and subjectively. Starts in sleep and springs out of bed, throws off bed-clothes, tears her dress, lies naked and resists covering. Heart-sounds intermit, shocks with both sounds. Eats nothing. Pulse 94. *Rhus tox.* CM, in water, every three hours, then every six hours.

Dec. 22d. *Rhus* was given until yesterday, when general perspiration appeared, offensive, sour, with relief of pain (ate fruit, and gruel for first time), slept quietly. (Fourteenth day. Sour sweat as heretofore, three attacks, announced relief about the eighth week).

1881, Jan. 3d. Weeps often. For five days, flatulent sour stomach. *Soles very sore* for weeks past. *Pulsat.* CM, in water, twice, six hours apart. This seemed to have been efficient.

V. 1880, June 6th. Mrs. A. F. S., æt. 66. While suffering from acute articular rheumatism, three years ago, *and under "homœopathic treatment,"* an eruption coming out over the knee-joints was suppressed by carbolic acid.

In bed or on crutches since, with bony outgrowths at extremities of femurs and their articulates, especially left knee; also, right elbow and right hand, left knee the larger, with its pronator ligaments considerably shortened. Pains in the exostoses were worse after rest, early in the morning, and from changes of temperature. Sweat all over, during severe pains. Rubbing quiets the pains, which are darting, numb, sore (not increased in damp weather). Has much coccygeal pain, end of coccyx sensitive since parturitions. Prolapsus, weakness of vesica, and frequent micturition. Pruritus, altogether feeble.

*Rhus tox.*, *Graphit.*, *Merc. sol.*, *Pulsat.*, *Bryonia*, *Silicea* and *Calc. fluorat.*, each CM, were given about a month apart, with little noteworthy effect; when *Sulph.* DM, given Dec. 31st (five weeks after *Cal. fluor.*), led (Feb. 8th), to report of considerable improvement. *An eruption of urticaria appeared at this time.* Mrs. S. now made a short journey without serious inconvenience. The stiffness and periosteal enlargement, as must be supposed, were not materially changed, yet the relief of her progressive deformity and its attendant pains, unchecked during three years, is reasonably satisfactory considering the nature of the case.

